# Influence of connection configuration on the punching resistance of CFST column–RC slab systems under eccentric loading

**DOI:** 10.1038/s41598-026-46159-9

**Published:** 2026-04-16

**Authors:** Mohamed Ghalla, Rabeea W. Bazuhair, Yahya M. Bin Mahfouz, Moataz A. Badawi, Ramy I. Shahin, Mohamed A. Altobgy

**Affiliations:** 1 Civil Engineering Department, Faculty of Engineering, Kafrelsheikh University, Kafrelsheikh, Egypt; 2 Civil Engineering Department, College of Engineering and Architecture, Umm Al-Qura University, Makkah, Saudi Arabia; 3 Department of Civil Engineering, Higher Institute of Engineering and Technology, Kafrelsheikh, Egypt

**Keywords:** CFST column, Reinforced concrete slab, Punching shear, Eccentric loading, Welded bars, Bolted connection, Engineering, Materials science

## Abstract

This study investigates the punching shear behaviour and failure mechanisms of concrete-filled steel tube (CFST) column reinforced concrete (RC) slab connections subjected to eccentric loading through an integrated experimental and numerical approach. Twelve specimens with different connection details, including welded bars, bolted connections, and C-shaped embedded bars, were tested to assess the influence of connection configuration, reinforcement arrangement, and bolt embedment length on structural performance. The experimental results showed that welded and bolted connections markedly enhanced cracking load, ultimate capacity, and ductility compared with the control specimen, while the combined welded–bolted configuration achieved the most favourable overall response. The study enhances the understanding of load transfer mechanisms in CFST–RC slab connections. Incorporation of welded bars and bolts substantially enhanced the connection stiffness, strength, and ductility. The two-row welded bar configuration showed significant improvements in load-carrying capacity, while the hybrid welded–bolted connection demonstrated the highest overall performance.

## Introduction

The construction industry has witnessed a significant paradigm shift toward hybrid structural systems that combine the advantages of different materials while mitigating their individual limitations^1–6^. Among these innovative solutions, concrete-filled steel tube (CFST) columns coupled with reinforced concrete (RC) flat slab systems have emerged as a particularly promising structural framework for high-rise construction projects^7–10^. This composite structural approach represents a synthesis of steel’s tensile strength and ductility with concrete’s compressive capacity and fire resistance, creating a synergistic system that addresses the evolving demands of modern construction^11^.

The adoption of CFST columns in tall building projects has been driven by their superior structural performance characteristics, including enhanced axial strength capacity, increased structural rigidity, improved seismic energy dissipation, and expedited field installation processes^12,13^. The composite action between the steel exterior and concrete core creates unique mechanical properties that overcome the individual material limitations while amplifying their respective strengths. In this system, the outer steel tube provides lateral confinement to the concrete core, enhancing its mechanical properties and load-bearing capacity while simultaneously resisting tensile and shear forces. Conversely, the internal concrete prevents inward buckling of the steel shell and efficiently carries compressive loads^14^.

The integration of CFST columns with RC flat slab systems presents particular advantages in terms of architectural flexibility and construction efficiency. Flat slab configurations facilitate the reduction of floor-to-floor heights, which is crucial in tall structures where traditional beam-supported slabs consume valuable vertical space. Furthermore, these systems provide greater flexibility for spatial arrangements and building services integration while simplifying construction processes. However, the successful implementation of CFST-flat slab systems critically depends on the development of efficient and reliable connection details between the steel tube column and the concrete slab^15^.

Extensive research has addressed the challenge of connecting CFST columns to RC flat slabs, focusing on constructability and structural integrity. A significant contribution was made through the development of a connection where the steel tube is strategically cut at the slab level to prevent separation between the slab and the concrete inside the steel tube, with the joint zone strengthened using rebar rings and longitudinal reinforcement^16^ Seismic performance evaluation has been a primary research focus, particularly for buildings composed of coupled shear walls with dampers, CFT columns, and flat plate slabs, which demonstrate potential for reuse with low repair costs following seismic events^17^. Full-scale experimental investigations under gravity loading conditions have demonstrated that properly designed CFT-RC connections can exhibit punching shear strength and connection stiffness exceeding those of conventional RC flat plate systems^18^. Semi-analytical procedures have been developed to model connection behavior from elastic to post-punching ranges, with applications in progressive collapse analysis. Innovation in connection design has extended to novel ductile structural steel shear head systems that maintain gaps around columns, enabling slab connections through isolated steel members designed to yield in shear before punching failure^19^. These systems demonstrate enhanced ductility and energy dissipation under combined gravity and cyclic lateral loading.

Advanced analytical approaches using finite element analysis have been employed to examine stress concentration, load flow, and deformation patterns in CFST-slab connections^15^. Parametric studies analyzing steel tube dimensions, wing plate characteristics, steel strength, and reinforcement eccentricity have resulted in validated design equations. Research on hybrid members consisting of RC flat slabs connected to steel columns through fully integrated shear-heads has provided comprehensive data on load-deformation behavior and ultimate punching shear strength^20^. These investigations have established analytical models for rotational response and flexural strength based on shear-head characteristics and led to specialized design expressions.

Prefabricated connection systems have addressed both seismic performance and constructability concerns through novel connections between prefabricated RC flat slabs and square steel tube columns^21^. These systems feature controlled plastic deformation in replaceable cantilever beams and have demonstrated superior seismic performance in experimental and numerical investigations. Advanced connection concepts utilizing steel plate shear-heads have been validated through large-scale experiments and numerical simulations, resulting in analytical prediction models for punching shear capacity^22^.

The application of unbonded post-tensioned concrete (UPC) slabs in CFST connections represents an emerging area with significant potential^23^. Experimental investigations have shown that UPC slab-CFT column specimens using innovative connections exhibit superior punching shear resistance (up to 10% increase), ultimate deformation capacity (up to 39% increase), and energy absorption capacity (up to 25% increase) compared to conventional systems.

High-performance concrete integration in critical punching shear zones has emerged as an effective method for improving connection performance^24^. Experimental programs utilizing engineered cementitious composite (ECC) and ultra-high performance ECC (UHPECC) have achieved ultimate load increases of up to 33% and 28% for concentric and eccentric loading conditions, respectively, while improving cracking load, elastic stiffness, energy absorption capacity, and ductility. Improving the connection performance at the design stage is generally more effective than relying on strengthening techniques proposed in previous studies^25–27^, such as using high-performance concrete or sustainable strengthening materials^28–31^. Although these methods can enhance structural capacity, they are often applied after construction, which may increase cost and complexity. In contrast, optimizing the connection configuration from the beginning improves load transfer, stiffness, and crack resistance without the need for additional strengthening measures, making it a more efficient and practical approach for enhancing the punching resistance of CFST column–RC slab systems under eccentric loading.

Novel approaches involving steel skeleton embedment have been developed to enhance punching shear capacity and ductility^32^. Experimental investigations examining steel skeleton type, reinforcement ratio, steel content, and slab thickness have demonstrated effective improvement in punching shear capacity while increasing structural ductility by changing failure modes from punching shear to bending punching. Recent research has addressed the significant influence of load eccentricity on punching shear capacity, revealing that increasing eccentricity causes crack formation to shift toward higher loaded sides with incompletely developed punching cones^33^, similar systematic investigations for CFST column connections under eccentric loading conditions remain limited in the literature.

While the current body of research demonstrates the viability and advantages of CFST-slab systems, several critical research gaps remain unaddressed. Although recent studies have begun to examine the influence of load eccentricity on punching shear behavior^33^, the study focus on columns made of reinforced concrete. In addition, most investigations have focused primarily on concentric loading conditions, leaving a significant knowledge gap regarding the performance of various connection configurations under eccentric loading scenarios. Furthermore, while different connection types have been explored individually, limited comparative studies exist that systematically evaluate multiple connection configurations of CFST columns under identical loading conditions to identify optimal solutions for specific applications.

The proposed study addresses the research gaps discussed in the literature by conducting a comprehensive experimental study on the punching performance of reinforced concrete slabs connected to CFST columns under eccentric loading conditions. The research innovation lies in the systematic comparison of three distinct connection configurations: bolted connections, welded bar connections, and C-shaped bar connections, evaluated under identical eccentric loading protocols. This comparative approach provides unprecedented insights into the relative performance characteristics of different connection types under realistic loading conditions that commonly occur in practice but have received limited attention in existing literature.

The scope of the current study covers the experimental evaluation of punching shear behavior, load-deformation characteristics, failure modes, and energy dissipation capacity of each connection type under eccentric loading. Innovation and Scope of Current Study. This research aims to provide performance evaluation, identify optimal connection strategies for eccentric loading scenarios, and develop design recommendations that enhance the practical application of CFST-slab systems in real-world construction projects.

## Experimental program

### Specimen details

**Fig. 1:**
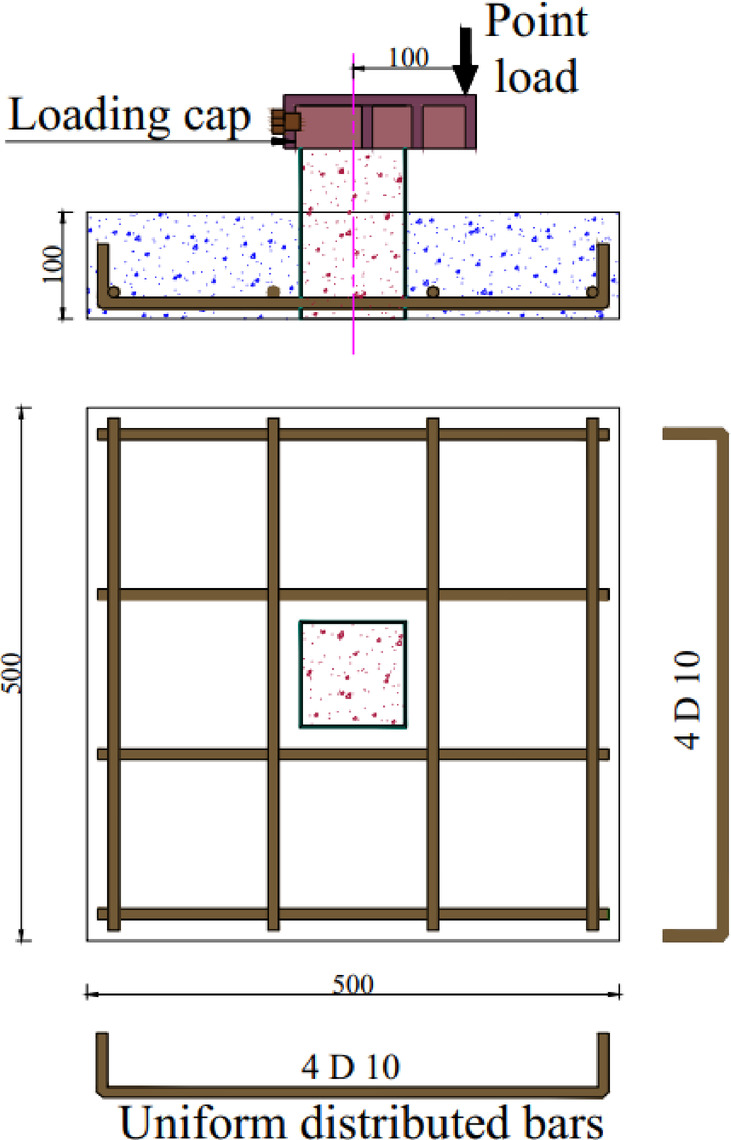
The geometric and reinforcement details for the tested slabs. (Dimension in mm)

**Fig. 2:**
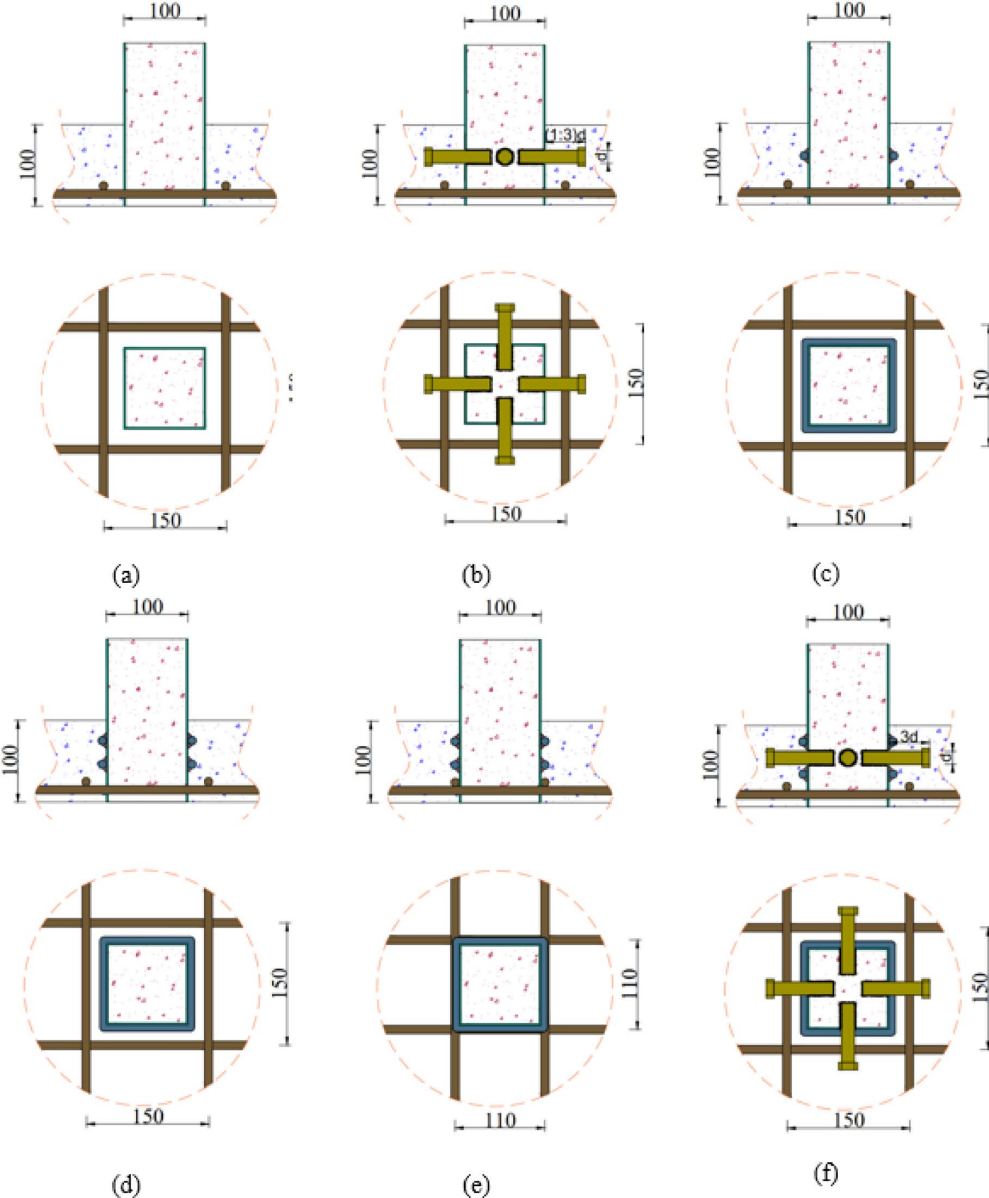

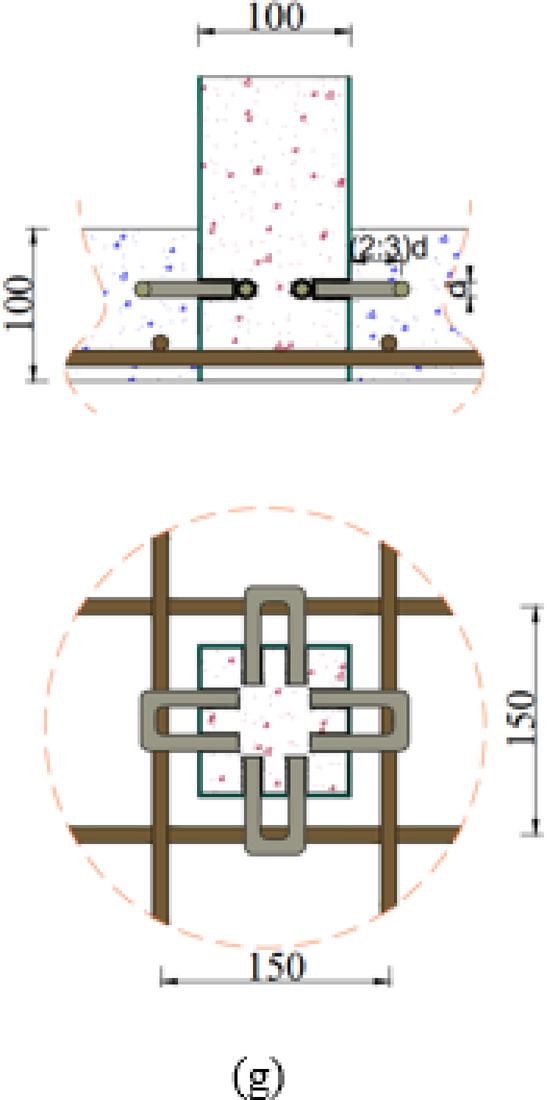
Arrangement and configuration of tested slabs: (**a**) columns without any preparations, (**b**) columns with bolts, (**c**) columns with welded bars, (**d**) columns with two welded bars, (**e**) nonuniform distribution of reinforcement bars, (**f**) columns with welded bars and bolts, and (**g**) columns with c-shape bars. (Units: mm)

The experimental program comprises six groups (G1-G6) with a total of twelve specimens designed to investigate various connection configurations between CFST columns and RC slabs under eccentric loading conditions, as shown in Table 1. The slab has a square section with 500 mm length, 100 mm in depth, and reinforced with four bars D10 in both direction at bottom, as illustrated in Fig. 1. Group G1 serves as the control group, containing specimen S (master slab without connection) to establish baseline performance, as illustrated in Fig. 2(a). Group G2, as illustrated in Fig. 2(c), investigates welded steel bar connections around columns, including specimens S-1WR8 (one welded row with D8 bars) and S-1WR10 (one welded row with D10 bars). Group G3 extends the welded bar investigation with specimens featuring two welded rows: S-2WR10 (two welded rows D10) as illustrated in Fig. 2(d), S-2WR10-N (similar to S-2WR10 but with slab reinforcement positioned near welded rows) as illustrated in Fig. 2(e), and S-2WR10-A3 (similar to S-2WR10 plus 4 bolts), as illustrated in Fig. 2(f).

Group G4 investigates the embedded length effect of bolts through specimens S-A1, S-A2, and S-A3 with embedded lengths of 16 mm, 32 mm, and 48 mm, respectively, as illustrated in Fig. 2(b). Group G5 examines different variables while maintaining the same bolt embedded length (48 mm) based on specimen S-A3 configuration. This group includes S-A3-W (4 bolts plus wide reinforcement), S-A3-M (4 bolts plus steel mesh), and S-A3-D (4 bolts plus upper reinforcement). Group G6, as illustrated in Fig. 2(g), investigates C-shaped connections with different embedded lengths, containing specimens S-C16-A2 (embedded length A2 = 24 mm) and S-C16-A3 (embedded length A2 = 48 mm), as shown in Table 1.

**Table 1:** Test matrix.

Group	Slab`s ID	Studied parameter	Used elements	Embedded length
G1	S	Master slabs	–	–
G2	S	Diameter of welded steel bars around columns	–	–
	S-1WR8		One welded row	Bar diameter = 8 mm
	S-1WR10			Bar diameter = 10 mm
G3	S	Welding steel bars around columns	–	–
	S-2WR10		Two welded rows (Bar diameter = 10 mm)	Bar diameter = 10 mm
	S-2WR10-N			Near reinforcement
	S-2WR10-A3		Two welded rows + 4 bolts	Bar diameter = 10 mm + 48 mm length of bolts
G4	S	Embedded length of bolts	–	–
	S-A1		4 bolts	A1 = 16 mm
	S-A2			A2 = 32 mm
	S-A3			A3 = 48 mm
G5	S	Different variables with the same embedded length of bolts	–	–
	S-A3-W		4 bolts + wide reinforcement	A3 = 48 mm
	S-A3-M		4 bolts + steel mesh	
	S-A3-D		4 bolts + upper reinforcement	
G6	S	C-shape with different embedded length	–	–
	S-C16-A2		C-shape	A2 = 24 mm
	S-C16-A3			A3 = 48 mm

WR: welded Row; WR8/10: welded Row with 8/10 mm diameter; N: Near reinforcement; A (1:3): Anchored length of bolts; W: Wide reinforcement; M: welded steel mesh; D: Double reinforcement layers; C: The shape of embedded bars.

### Studied parameters

The experimental investigation systematically examines several key parameters that influence the punching shear performance of CFST-slab connections under eccentric loading. The primary parameters include connection type, with three main categories investigated: welded steel bar connections, bolt connections, and C-shaped connections. For welded steel bar connections, the study evaluates the effect of bar diameter (D8 vs. D10), number of welded rows (single vs. double rows), and the interaction between welded bars and bolt reinforcement. The bolt connection investigation focuses on embedded length effects, examining three different embedment depths (16 mm, 32 mm, and 48 mm) to determine optimal anchor performance. Additional parameters for bolt connections include the influence of reinforcement configuration, specifically wide reinforcement distribution, steel mesh integration, and upper reinforcement placement. For C-shaped connections, the study investigates embedded length variations (24 mm vs. 48 mm) to assess their impact on connection performance.

Secondary parameters include slab reinforcement positioning relative to welded rows, examining how proximity affects load transfer mechanisms and punching shear capacity. The combined effect of different connection systems (welded bars plus bolts) is also investigated to evaluate potential synergistic benefits. These parameters are systematically varied to provide comprehensive insights into the relative importance of each factor on connection performance, failure modes, and overall structural behavior under eccentric loading conditions.

### Material properties and mix proportion

The experimental program utilized normal strength concrete consisting of sand as fine aggregate, crushed dolomite as coarse aggregate, and tap water. The detailed mix proportions and corresponding concrete compressive strength of 30 MPa are presented in Table 2. In addition to compressive strength, the tensile properties of the concrete were evaluated using splitting tensile tests on standard cylindrical specimens at 28 days, the average splitting tensile strength was about 3.2 MPa.

The mechanical properties of the reinforcing steel bars, steel CFST columns, steel bolts, and C-shaped bars used in the connection systems are summarized in Table 3. High-strength bolts were employed for the bolt connection configurations to ensure adequate tensile and shear capacity for load transfer between the CFST columns and RC slabs.

**Table 2:** Concrete mix component and mechanical properties.

Concrete	Cement (kg/m^3^)	Fine aggregate (kg/m^3^)	Coarse aggregate (kg/m^3^)	Water/binder	f_c_` (MPa)	F_t_ (MPa)
NC	410	720	1190	0.41	30	3.2

**Table 3:** Steel properties for used elements.

Material	Yield		Ultimate		E (MPa)
	σ_y_ (MPa)	$\epsilon$_y_ (%)	σ_u_ (MPa)	$\epsilon$_u_ (%)	
Steel bars for reinforcement	349	1.59	611	13.01	219
Steel bolts for connectors	406	1.86	725	10.29	218
Steel sections for CFST columns	425	1.89	732	11.25	225

### Casting and preparation

**Fig. 3:**
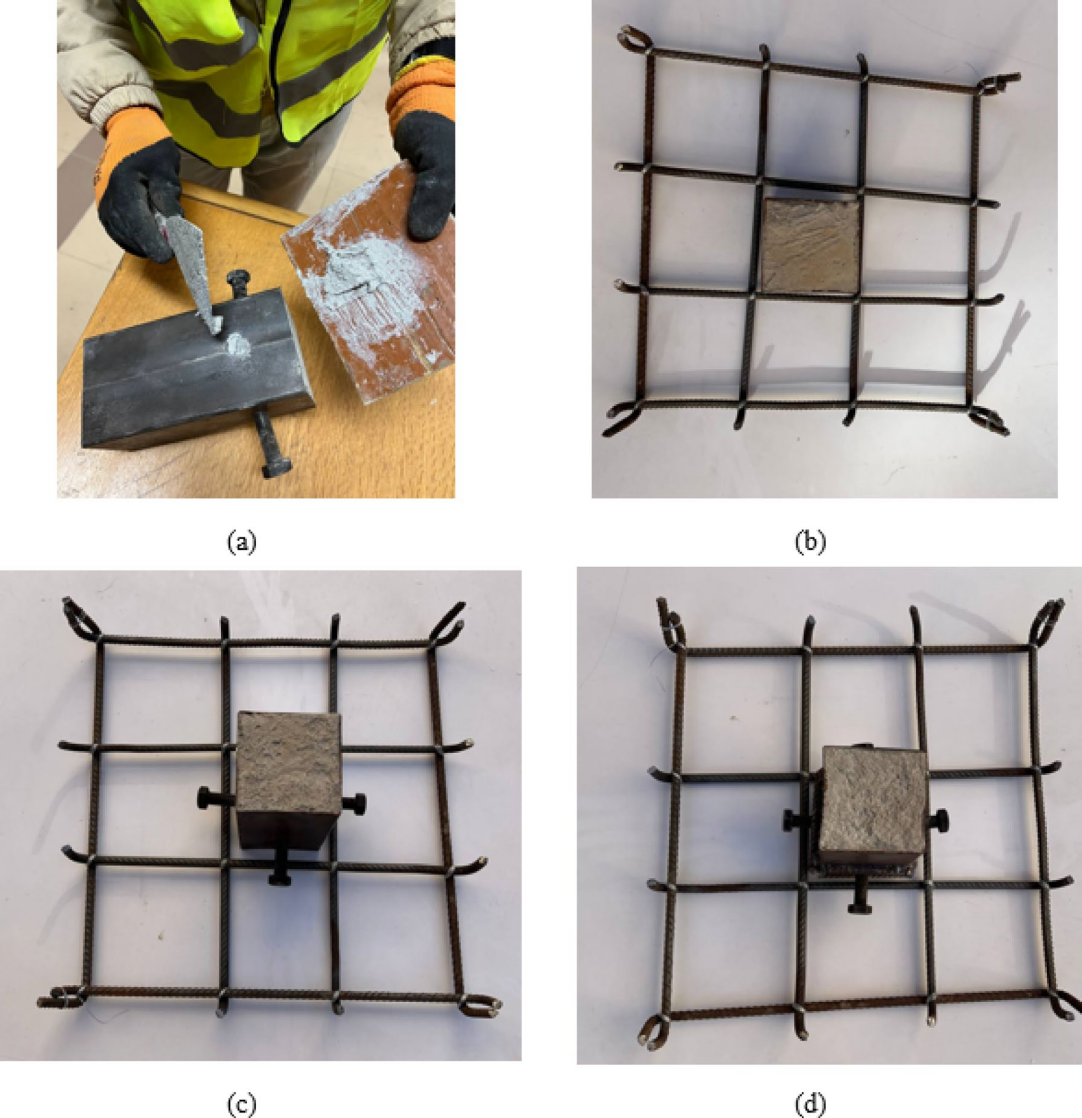
Configuration of columns with connections: (**a**) installation of bolts, (**b**) columns without any preparations, (**c**) columns with bolts, (**d**) columns with welded bars and bolts.

The specimen preparation process involved a sequential casting procedure to ensure proper integration between the CFST columns and RC slabs. Initially, the CFST columns were cast and allowed to cure for 7 days under standard curing conditions. Following the initial curing period, the connection elements were prepared according to the specific requirements of each specimen group. For the control specimen S, the cured CFST column was positioned at the center of the slab formwork without any additional connection preparation, as illustrated in Fig. 3 (b, c, and d). For specimens requiring bolt connections, the bolts were securely attached to the CFST column using epoxy resin adhesive to ensure proper bonding and load transfer capability, as shown in Fig. 3 (a). Similarly, for welded bar connection specimens, the reinforcing bars were welded directly to the CFST column surface as depicted in Fig. 3 (d). After completing the connection element installation, the slab concrete was cast within the prepared formwork, completely encasing the CFST column and connection elements. The completed specimens were then subjected to a standard 28-day curing period to achieve the required concrete strength before testing.

For specimens incorporating welded bar connections, the reinforcing bars were attached to the external surface of the steel tube using continuous fillet welds. The weld thickness was 5 mm and was applied continuously along the full contact length of the bar with the steel tube from both the upper and lower sides to ensure adequate load transfer and connection rigidity. All welding operations were carried out using standard arc welding procedures by a qualified technician, and the welds were visually inspected to ensure proper continuity and quality.

For specimens utilizing bolt connections, the bolts were anchored to the steel tube using a two-component structural epoxy adhesive (SikaDur-31). The adhesive has a compressive strength of approximately 70–80 MPa, tensile strength of about 15–20 MPa, elastic modulus of approximately 4–6 GPa, and bond strength exceeding 14 MPa depending on substrate conditions. Prior to installation, the steel surfaces were mechanically cleaned and degreased to ensure proper adhesion. The epoxy components were mixed according to the manufacturer’s recommended ratio, applied to the prepared surfaces, and the bolts were positioned and held in place until the adhesive achieved its initial curing stage.

### Loading arrangement, test setup and instrumentation

**Fig. 4:**
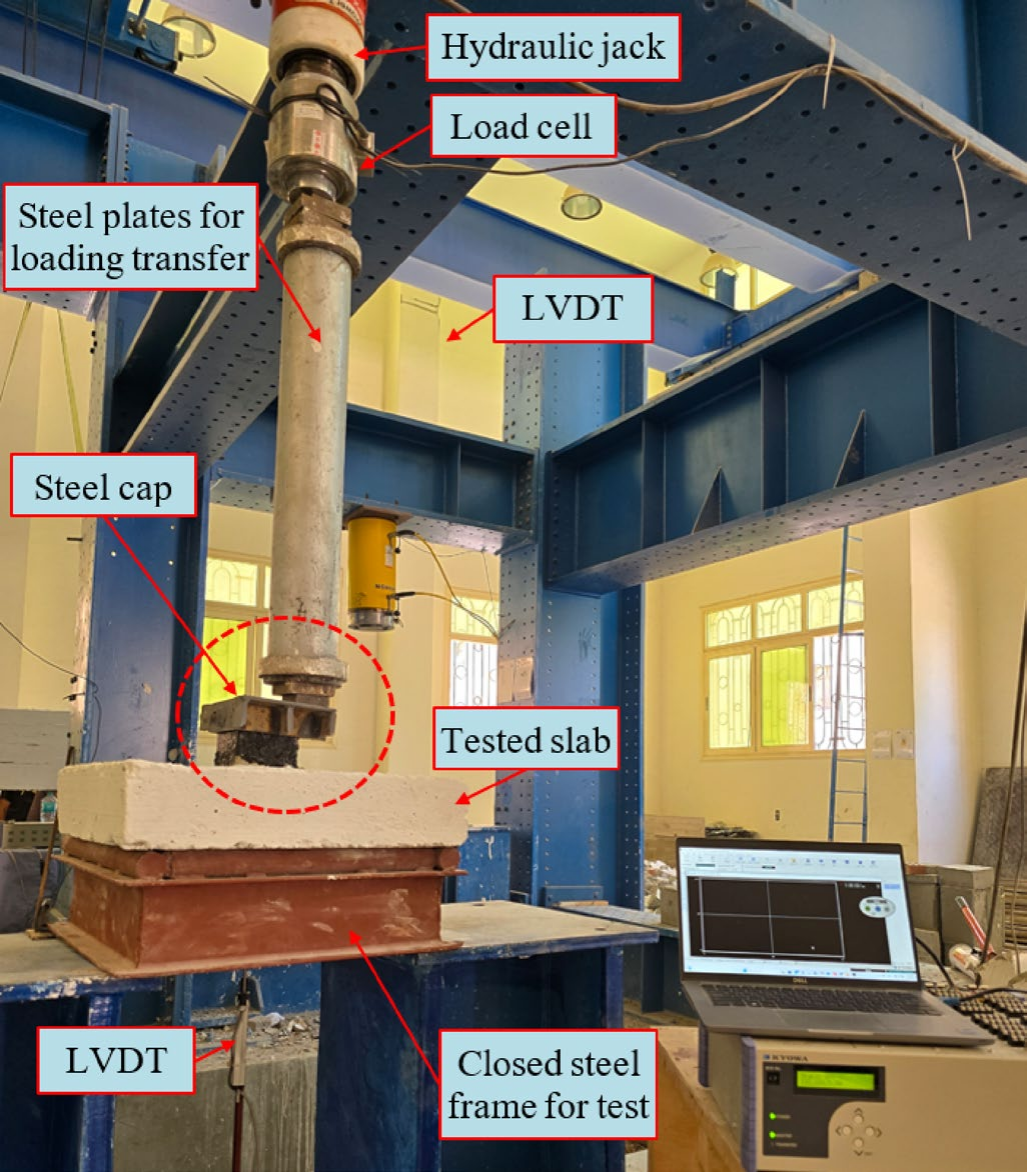
Test set-up and instrumentation of the tested slab.

The experimental investigation was conducted at the Concrete Laboratory of Kafrelsheikh University. A self-reacting steel frame with high stiffness was utilized to provide the necessary support for the testing of the slab specimens, as illustrated in Fig. 4. Each concrete slab was positioned and leveled on the lower supports of the steel frame. The slab specimens were supported along their edges using steel supports designed to provide vertical reaction while permitting limited rotational movement. Accordingly, the boundary condition may be described as approximately simply supported with partial rotational restraint due to friction and contact stiffness at the support interfaces. This support configuration was maintained consistently for all specimens to ensure comparable load distribution and punching response under eccentric loading. The setup was designed to ensure that the boundary conditions were consistent across all tested specimens. The load was applied vertically at 100 mm from the center of the slab to simulate an eccentric column load and induce a punching shear failure mode. The loading sequence was controlled by a servo-hydraulic actuator (hydraulic jack), which was mounted to the upper crossbeam of the reaction frame.

Vertical displacement was measured using linear variable differential transformers (LVDTs) positioned at selected locations on the slab surface. One LVDT was installed directly beneath the loading point to record the primary vertical deflection, while additional LVDTs were placed symmetrically around the slab to monitor global deformation and potential rotation.

Electrical resistance strain gauges were installed on selected reinforcement bars near the column–slab interface and, where applicable, on connection elements to monitor strain development in critical regions. The location and number of strain gauges were selected to capture the response of the connection zone where stress concentration and cracking were expected to occur. Crack development was monitored visually throughout the loading process. The slab surface was pre-marked with a white colour to facilitate crack detection and propagation tracking. The first cracking load was defined as the load corresponding to the first visible surface crack observed by direct visual inspection, confirmed by a sudden change in the load–deflection response or local stiffness reduction. Crack patterns and propagation were recorded at regular load intervals until failure.

The applied load was transferred from the actuator through a calibrated load cell to a series of thick steel plates and a transfer column. These plates ensured a uniform distribution of the eccentric load onto a rigid steel cap. The steel cap was placed directly on the top surface of the concrete slab, serving as the loading point. This arrangement was designed to apply an eccentric load over a defined area, as illustrated in Figs. 1 and 4.

## Test results and discussion

### Crack pattern, cracking load, and ultimate load

**Fig. 5:**
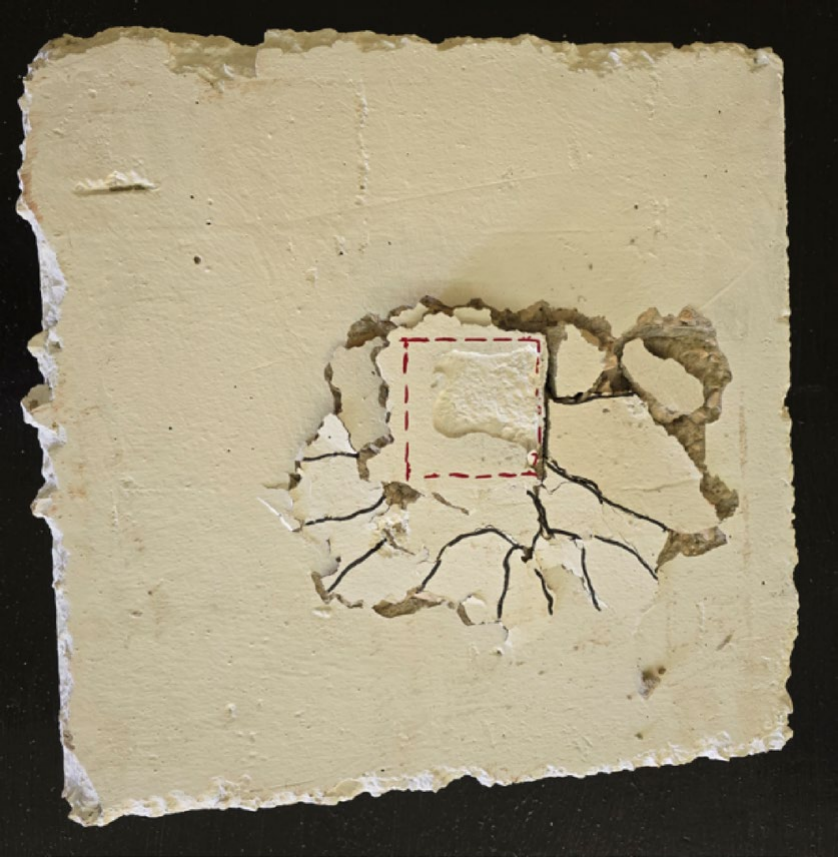
Crack pattern of master slab (S).

The experimental investigation revealed significant differences in the structural behavior and failure patterns between the control specimen and specimens with welded bar connections. The master slab specimen S, without any connection enhancement, exhibited the lowest structural performance with a cracking load of 2.92 kN and an ultimate load capacity of 13 kN, as illustrated in Table 4. As shown in Fig. 5, the crack pattern of specimen S demonstrated a typical punching shear failure characterized by extensive radial cracking emanating from the column perimeter. The crack propagation formed a concentrated punching cone around the CFST column interface, with significant concrete spalling observed at the failure zone, indicating a brittle failure mode with limited ductility and energy dissipation capacity.

**Fig. 6:**
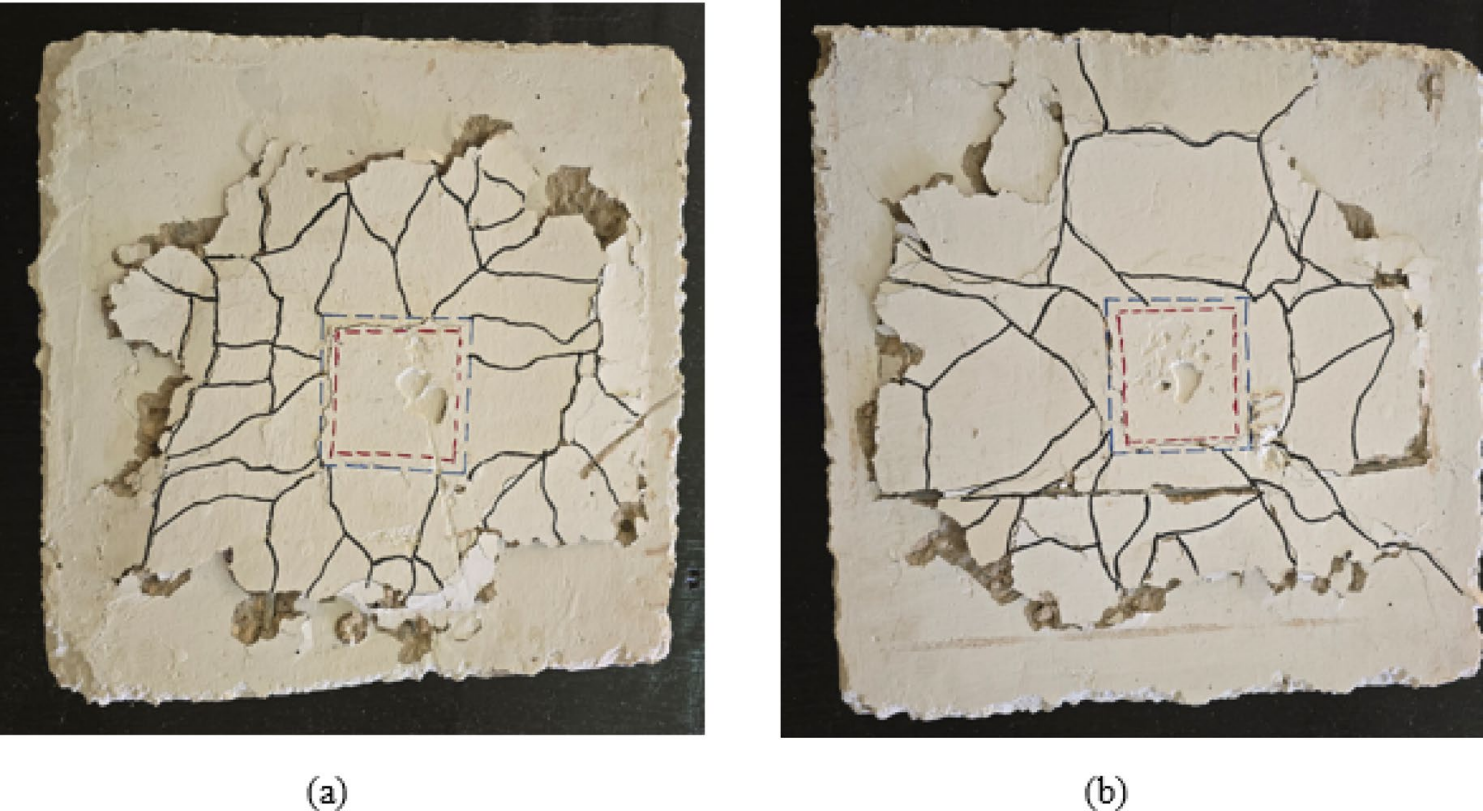
Crack pattern of group G2: (**a**) slab S-1WR8 and (**b**) slab S-1WR10.

In contrast, specimens with welded bar connections in Group G2 demonstrated substantially improved structural performance and altered failure mechanisms. Specimen S-1WR8, incorporating one welded row of D8 bars, achieved a cracking load of 8.1 kN and an ultimate load of 21 kN, representing increases of 177% and 62% respectively compared to the control specimen, as illustrated in Table 4. The crack pattern shown in Fig. 6(a) reveals a more distributed cracking behavior with radial cracks extending across a larger slab area. The welded bars effectively transferred loads from the slab to the CFST column, resulting in more gradual crack development and improved post-cracking performance. The failure pattern exhibited characteristics of combined punching-flexural behavior rather than pure punching shear failure. Specimen S-1WR10, featuring one welded row of larger diameter D10 bars, demonstrated further performance enhancement with a cracking load of 10.32 kN and an ultimate load of 26 kN, representing increases of 253% and 100% respectively compared to specimen S, as illustrated in Table 4. As illustrated in Fig. 6(b), the crack pattern shows a well-distributed network of radial and tangential cracks extending throughout the slab area. The larger bar diameter provided enhanced connection stiffness and load transfer efficiency, resulting in a more ductile failure mode. Notably, the crack distribution around the column interface was more uniform, with reduced concrete spalling compared to the control specimen, indicating improved stress distribution and connection integrity.

**Fig. 7:**
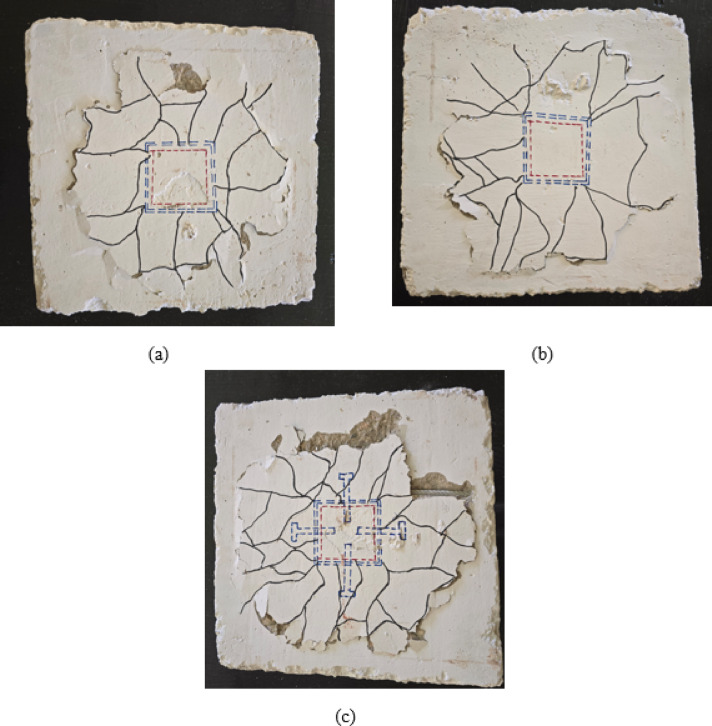
Crack pattern of group G3: (**a**) slab S-2WR10, (**b**) slab S-2WR10-N, and (**c**) slab S-2WR10-A3.

Group G3 specimens, featuring two welded rows of D10 bars, demonstrated progressive performance improvements through various configuration modifications. Specimen S-2WR10, with two welded rows of D10 bars, achieved a cracking load of 13.21 kN and an ultimate load of 38 kN, representing substantial increases of 352% and 192% respectively compared to the control specimen S, as depicted in Table 4. As illustrated in Fig. 7(a), the crack pattern exhibits a complex network of radial cracks with improved distribution around the column perimeter. The dual-welded rows enhanced load transfer capacity and provided more effective confinement to the connection zone, resulting in a wider crack distribution and reduced crack concentration near the column interface.

Specimen S-2WR10-N, with slab reinforcement positioned near the welded rows, demonstrated further enhancement with a cracking load of 14.61 kN and an ultimate load of 45 kN, representing increases of 10% and 18%, respectively, compared to S-2WR10, as depicted in Table 4. The crack pattern shown in Fig. 7(b) reveals a more controlled crack propagation with better integration between the slab reinforcement and welded connection bars. The strategic positioning of slab reinforcement near the welded rows improved the composite action between the connection elements and the surrounding concrete, resulting in enhanced stiffness and load-carrying capacity. The crack distribution demonstrates more uniform stress transfer throughout the slab, with reduced localized cracking around the column zone.

Specimen S-2WR10-A3, incorporating two welded rows of D10 bars plus four bolts, achieved the highest performance within Group G3 with a cracking load of 20.1 kN and an ultimate load of 56 kN, representing remarkable increases of 589% and 331% respectively compared to the control specimen, as shown in Table 4. As depicted in Fig. 7(c), the crack pattern shows extensive radial cracking distributed across a larger slab area with significant concrete spalling at multiple locations. The combination of welded bars and bolts created a hybrid connection system that effectively distributed stresses and delayed punching failure. The failure pattern indicates a transition toward a more ductile bending-dominated behavior, with the bolts providing additional anchorage and the welded bars ensuring effective load transfer. This hybrid configuration demonstrated superior performance by combining the advantages of both connection systems, resulting in enhanced ductility, energy dissipation capacity, and ultimate load-carrying capacity under eccentric loading conditions.

**Fig. 8:**
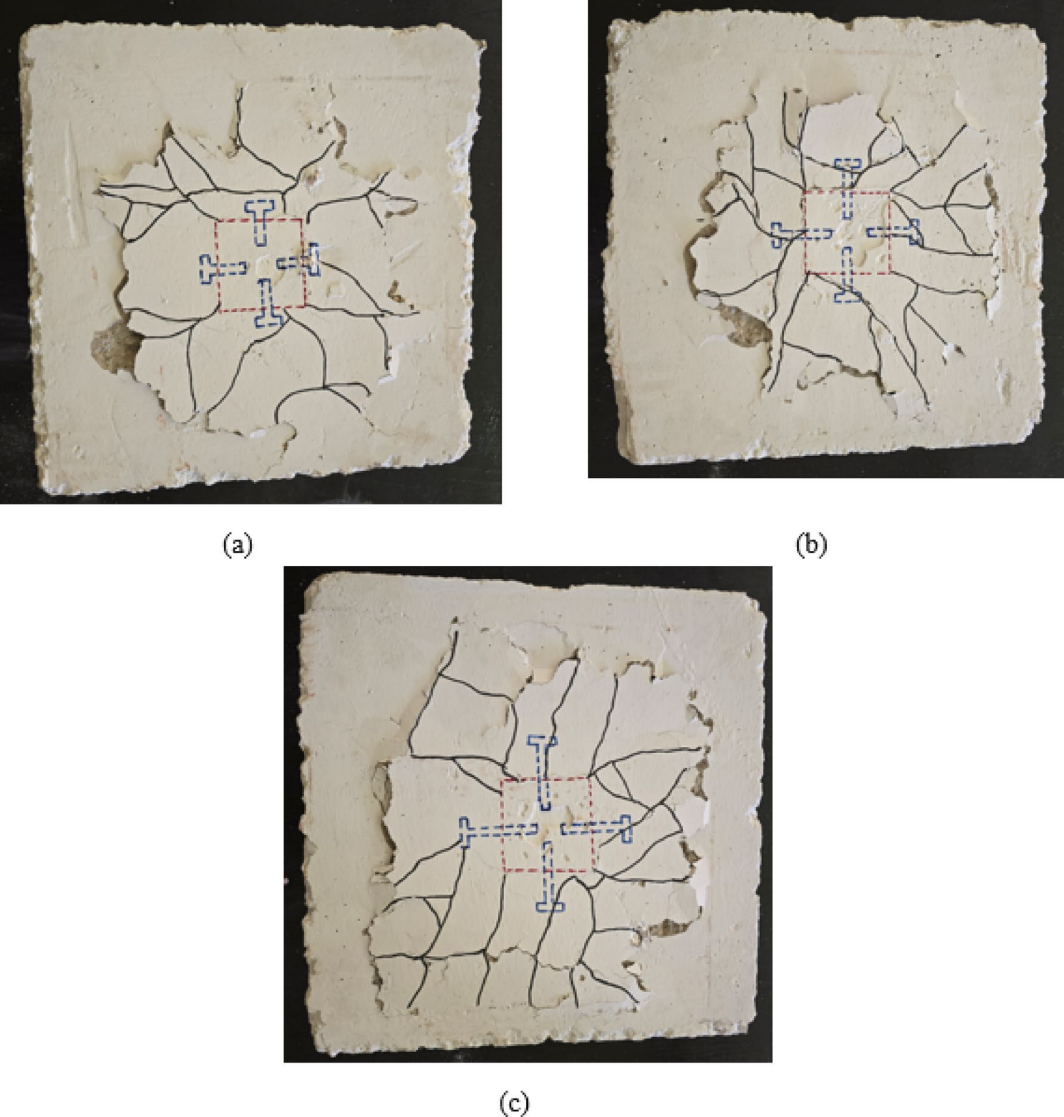
Crack pattern of group G4: (**a**) slab S-A1, (**b**) slab S-A2, and (**c**) slab S-A3.

Group G4 specimens investigated the influence of bolt embedded length on the punching shear performance of CFST-slab connections. Specimen S-A1, with four bolts having an embedded length of 16 mm, achieved a cracking load of 6.92 kN and an ultimate load of 20 kN, representing increases of 137% and 54% respectively compared to the control specimen S. As shown in Fig. 8(a), the crack pattern exhibits radial cracks emanating from the column perimeter with moderate distribution across the slab surface. The relatively short embedded length provided limited anchorage capacity, resulting in a crack pattern that shows some characteristics of punching failure with localized stress concentration around the bolt locations. The failure mode indicates that while the bolts improved performance compared to the control specimen, the shallow embedment depth restricted their effectiveness in distributing loads throughout the slab.

Specimen S-A2, with four bolts having an increased embedded length of 32 mm, demonstrated improved structural performance with a cracking load of 9.58 kN and an ultimate load of 27 kN, representing increases of 228% and 108% respectively compared to specimen S, and improvements of 38% and 35% over specimen S-A1, as depicted in Table 4. The crack pattern illustrated in Fig. 8(b) reveals a more distributed radial crack network extending across a larger slab area. The increased embedded length enhanced the anchorage effectiveness and load transfer mechanism, allowing better stress distribution between the bolts and surrounding concrete. The crack propagation shows reduced concentration near the column interface and more uniform stress distribution, indicating improved connection performance and delayed onset of punching failure.

Specimen S-A3, featuring four bolts with the maximum embedded length of 48 mm, achieved the highest performance within Group G4 with a cracking load of 11.47 kN and an ultimate load of 33 kN, representing substantial increases of 293% and 154% respectively compared to the control specimen, as illustrated in Table 4. As depicted in Fig. 8(c), the crack pattern displays well-distributed radial cracks with wider spacing and more uniform propagation throughout the slab. The maximum embedded length provided optimal anchorage capacity, enabling effective load transfer from the slab to the CFST column through the bolts. The failure pattern demonstrates a more ductile behavior with improved crack distribution and reduced localized damage around the connection zone. The results clearly indicate that increasing bolt embedded length from 16 mm to 48 mm progressively enhances both the cracking and ultimate load capacities, demonstrating the critical importance of adequate embedded length in achieving effective bolt connection performance under eccentric loading conditions.

To quantify the influence of key parameters on joint performance, the experimental results were evaluated using normalized performance indices relative to the control specimen. The analysis showed that punching shear capacity increased approximately linearly with bolt embedment length within the investigated range, indicating that anchorage depth is a dominant parameter governing load transfer efficiency. An empirical correlation between normalized ultimate capacity and normalized embedment length was established for the tested specimens. Similarly, increasing the number of welded bar rows and bar diameter produced measurable improvements in both ultimate load and ductility, with the number of welded rows demonstrating greater sensitivity than bar diameter within the tested configurations. The combined welded–bolt system exhibited the highest performance, indicating a synergistic interaction between mechanical anchorage and load redistribution. A comparative sensitivity assessment showed that connection configuration had the greatest influence on joint performance, followed by embedment length, while reinforcement arrangement provided secondary but measurable effects. These relationships represent experimental trends within the investigated parameter ranges and may support preliminary design assessment and connection optimization under eccentric loading.

**Fig. 9:**
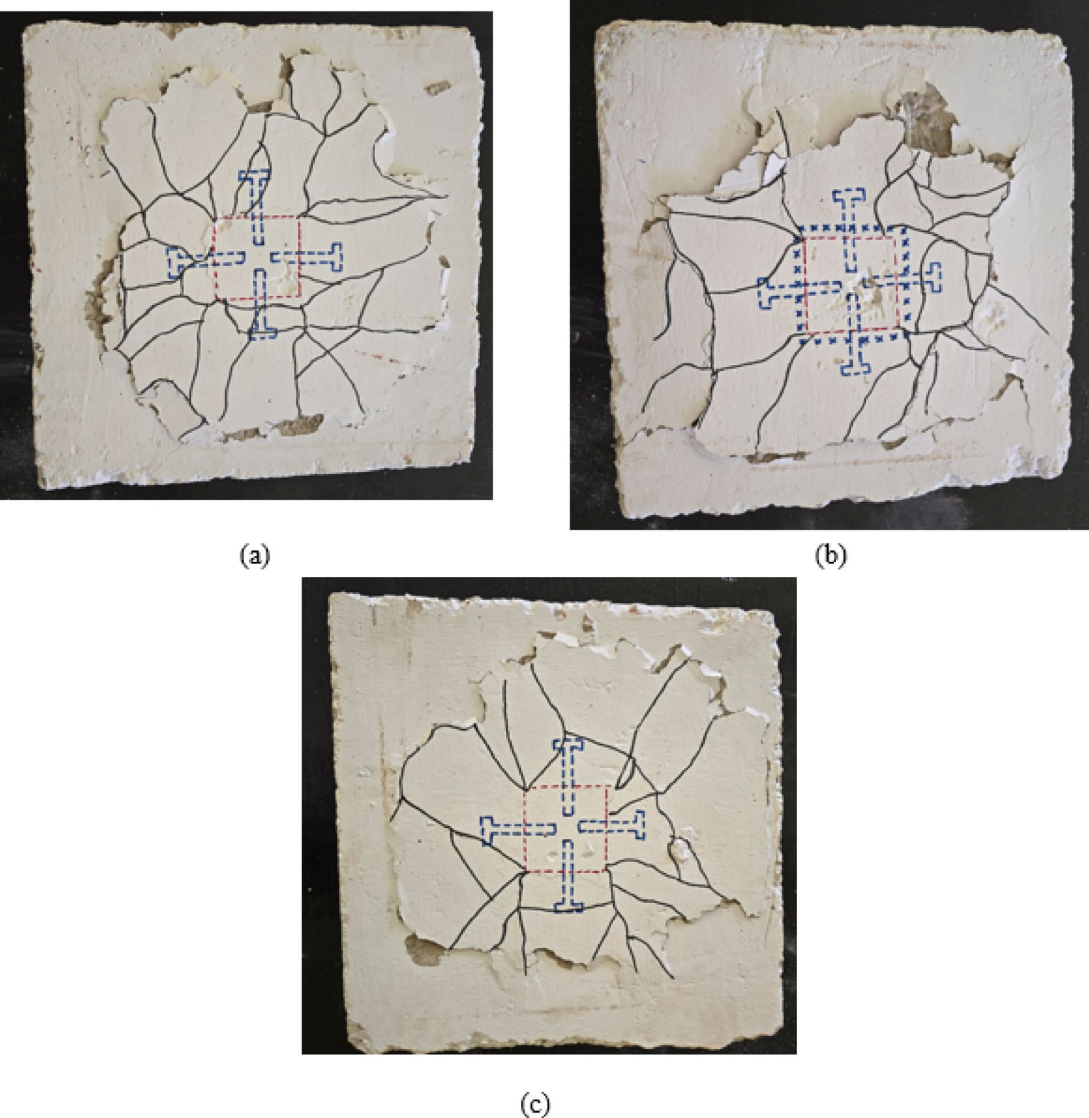
Crack pattern of group G5: (**a**) slab S-A3-W, (**b**) slab S-A3-M, and (**c**) slab S-A3-D.

Group G5 specimens examined the influence of different reinforcement configurations while maintaining a constant bolt embedded length of 48 mm based on the S-A3 configuration. Specimen S-A3-W, incorporating four bolts plus wide reinforcement distribution, achieved a cracking load of 9.14 kN and an ultimate load of 26 kN, representing increases of 213% and 100% respectively compared to the control specimen S, as depicted in Table 4. However, compared to the baseline specimen S-A3, S-A3-W showed decreases of 20% in cracking load and 21% in ultimate load capacity. As illustrated in Fig. 9(a), the crack pattern exhibits radial cracks with relatively wide spacing distributed across the slab surface. The wide reinforcement distribution, while intended to improve load distribution, appears to have reduced the local reinforcement density around the critical connection zone, resulting in earlier crack initiation and lower overall capacity. The failure pattern suggests that dispersing reinforcement over a wider area may compromise the concentration of resistance where it is most needed under eccentric loading conditions.

Specimen S-A3-M, featuring four bolts plus steel mesh reinforcement, demonstrated significantly improved performance with a cracking load of 13.52 kN and an ultimate load of 35 kN, representing substantial increases of 363% and 169% respectively compared to specimen S. Compared to the baseline S-A3, specimen S-A3-M showed improvements of 18% in cracking load and 6% in ultimate capacity, as depicted in Table 4. The crack pattern shown in Fig. 9(b) reveals a complex network of radial and tangential cracks with excellent distribution throughout the slab area. The steel mesh reinforcement provided enhanced two-dimensional resistance, effectively controlling crack propagation in multiple directions and improving the overall structural integrity of the connection zone. The extensive crack distribution with reduced crack widths indicates superior stress distribution and enhanced ductility. The mesh reinforcement effectively bridged cracks and maintained aggregate interlock, resulting in improved post-cracking behavior and energy dissipation capacity.

Specimen S-A3-D, incorporating four bolts plus upper reinforcement layer, achieved the highest performance within Group G5 with a cracking load of 14.18 kN and an ultimate load of 39 kN, representing remarkable increases of 386% and 200% respectively compared to the control specimen. Relative to specimen S-A3, S-A3-D demonstrated improvements of 24% in cracking load and 18% in ultimate capacity, as illustrated in Table 4. As depicted in Fig. 9(c), the crack pattern displays well-distributed radial cracks with controlled propagation and moderate crack widths extending across the slab surface. The additional upper reinforcement layer provided enhanced flexural resistance and improved moment capacity, which is particularly beneficial under eccentric loading conditions where significant bending moments develop. The failure pattern indicates a more ductile bending-dominated response with delayed punching failure. The upper reinforcement effectively resisted tensile stresses in the top fiber of the slab, resulting in superior load-carrying capacity and structural performance. The results demonstrate that strategic placement of additional reinforcement in the upper portion of the slab, combined with adequate bolt anchorage, represents an optimal configuration for enhancing connection performance under eccentric loading.

**Fig. 10:**
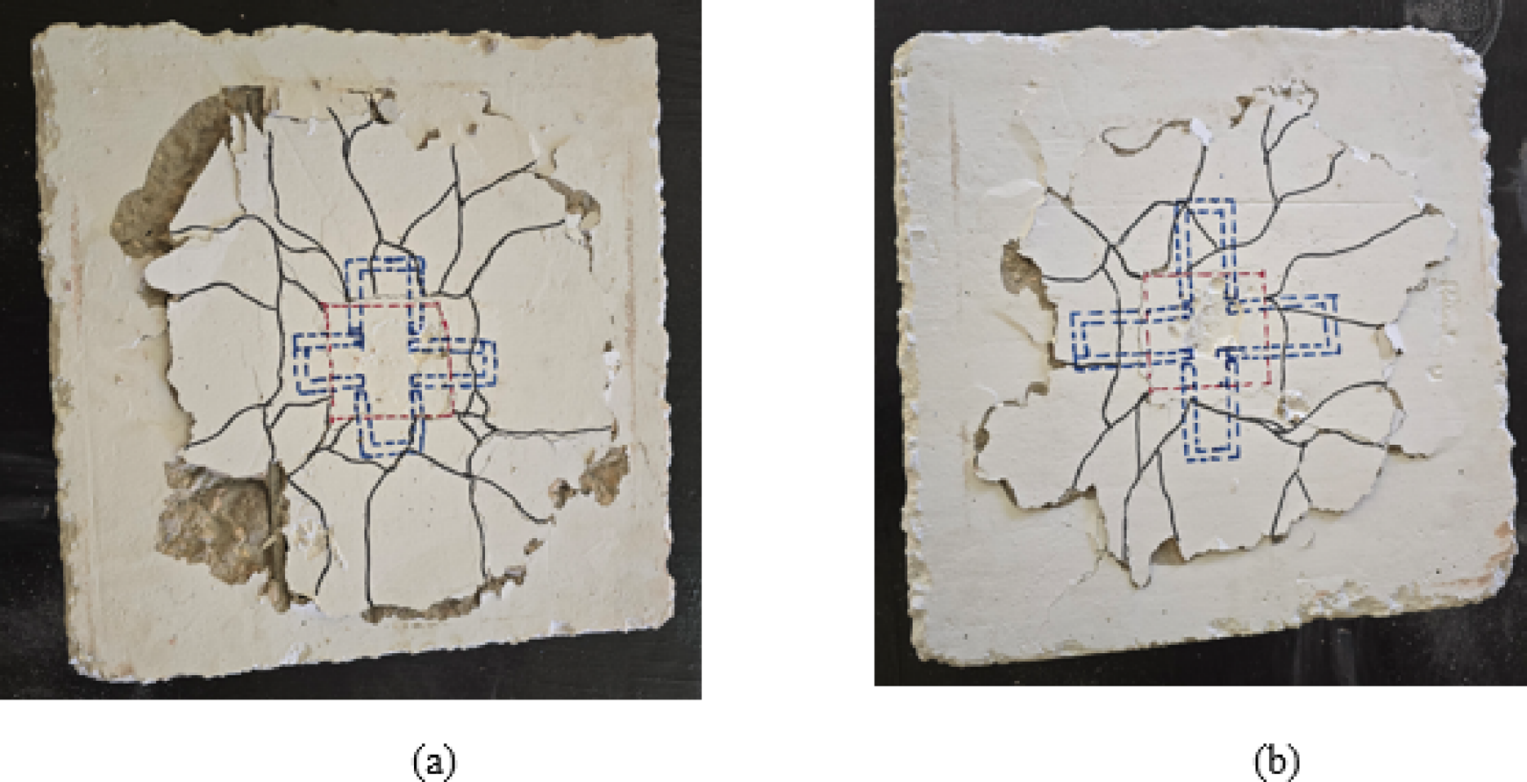
Crack pattern of group G6: (**a**) slab S-C16-A2 and (**b**) slab S-C16-A3.

Group G6 specimens investigated the performance of C-shaped connection configurations with different embedded lengths. Specimen S-C16-A2, featuring C-shaped bars with an embedded length of 24 mm (A2), achieved a cracking load of 14.82 kN and an ultimate load of 42 kN, representing substantial increases of 408% and 223% respectively compared to the control specimen S, as depicted in Table 4. As illustrated in Fig. 10(a), the crack pattern exhibits a distinct radial crack distribution with significant concrete spalling observed on one side of the specimen. The C-shaped connection provided effective mechanical anchorage and load transfer between the CFST column and the RC slab. However, the moderate embedded length of 24 mm resulted in somewhat asymmetric crack development, particularly evident in the concentrated damage zone on the left portion of the specimen. The failure pattern indicates that while the C-shaped bars effectively engaged with the surrounding concrete, the limited embedded length restricted their full potential in controlling crack propagation under eccentric loading, leading to localized concrete crushing and spalling in regions of high stress concentration.

Specimen S-C16-A3, incorporating C-shaped bars with an increased embedded length of 48 mm (A3), demonstrated the highest overall performance among all tested specimens with a cracking load of 15.88 kN and an ultimate load of 49 kN, representing remarkable increases of 444% and 277% respectively compared to the control specimen. Compared to specimen S-C16-A2, S-C16-A3 showed improvements of 7% in cracking load and 17% in ultimate capacity, as depicted in Table 4. The crack pattern shown in Fig. 10(b) reveals a well-developed radial crack network extending uniformly across the slab surface with more balanced crack distribution. The increased embedded length of 48 mm provided superior anchorage capacity and enhanced the mechanical interlocking between the C-shaped bars and the concrete matrix. The crack propagation demonstrates more symmetric behavior compared to S-C16-A2, indicating improved stress distribution and load transfer efficiency. The failure pattern exhibits characteristics of a more ductile response with extensive crack development before ultimate failure, demonstrating that the longer embedded length effectively mobilized the full capacity of the C-shaped connection system. The superior performance of specimen S-C16-A3 confirms that C-shaped connections with adequate embedded length represent a highly effective solution for connecting CFST columns to RC slabs under eccentric loading conditions, offering excellent load-carrying capacity, ductility, and energy dissipation characteristics.

**Table 4:** Test results of the tested slabs.

G	Specimen`s ID	Cracking Stage			Ultimate Stage			Elastic stiffness (K)	K/K_S_	Absorbed energy (E)	E/E_S_
		*P*_cr_ (kN)	*P*_cr_/*P*_crS_	Δ_cr_ (mm)	*P*_u_ (kN)	*P*_u_/*P*_uS_	Δ_Pu_ (mm)				
G1	S	2.92	1.00	0.36	10.36	1.00	1.90	8.11	1.00	17.78	1.00
G2	S	2.92	1.00	0.36	10.36	1.00	1.90	8.11	1.00	17.78	1.00
	S-1WR8	8.10	2.77	0.63	21.61	2.09	3.10	12.86	1.59	63.69	3.58
	S-1WR10	10.32	3.53	0.77	26.89	2.60	5.21	13.40	1.65	134.95	7.59
G3	S	2.92	1.00	0.36	10.36	1.00	1.90	8.11	1.00	17.78	1.00
	S-2WR10	13.21	4.52	0.89	38.42	3.71	4.60	14.84	1.83	176.13	9.91
	S-2WR10-N	14.61	5.00	0.73	45.79	4.42	4.81	20.01	2.47	244.82	13.77
	S-2WR10-A3	20.10	6.88	0.86	56.63	5.47	6.35	23.37	2.88	342.74	19.28
G4	S	2.92	1.00	0.36	10.36	1.00	1.90	8.11	1.00	17.78	1.00
	S-A1	6.92	2.37	0.63	20.73	2.00	4.17	10.98	1.35	76.75	4.32
	S-A2	9.58	3.28	0.79	27.6	2.67	5.39	12.13	1.50	152.61	8.58
	S-A3	11.47	3.93	0.81	33.58	3.24	6.23	14.16	1.75	207.67	11.68
G5	S	2.92	1.00	0.36	10.36	1.00	1.90	8.11	1.00	17.78	1.00
	S-A3-W	9.14	3.13	0.95	26.21	2.53	3.19	9.62	1.19	95.38	5.36
	S-A3-M	13.52	4.63	0.89	35.63	3.44	4.80	15.19	1.87	164.03	9.23
	S-A3-D	14.18	4.86	0.74	39.76	3.84	4.99	19.16	2.36	239.10	13.45
G6	S	2.92	1.00	0.36	10.36	1.00	1.90	8.11	1.00	17.78	1.00
	S-C16-A2	14.82	5.08	0.76	42.15	4.07	5.11	19.50	2.40	200.35	11.27
	S-C16-A3	15.88	5.44	0.61	49.38	4.77	5.22	26.03	3.21	274.83	15.46

P_cr_: Load at which the first crack appeared; Δ_cr_: Vertical deflection recorded at P_cr_; P_u_: Ultimate load; Δ_Pu_: Vertical deflection recorded at P_U_; K: elastic index; E: Absorbed energy.

### Load deflection response

**Fig. 11:**
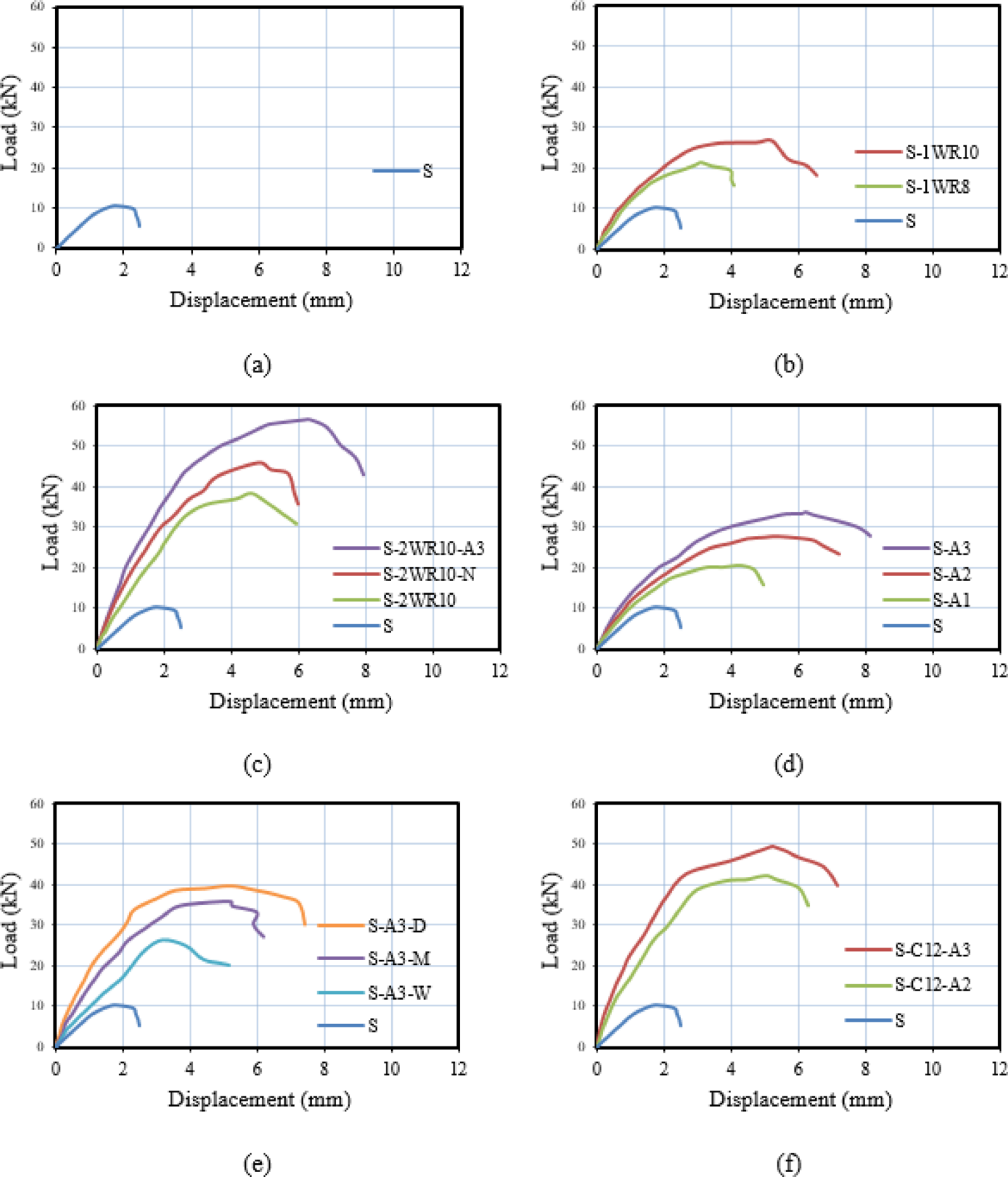
Load-deflection relationships for tested slabs: (**a**) Group G1, (**b**) Group G2, (**c**) Group G3, (**d**) Group G4, (**e**) Group G5, and (**f**) Group G6.

The load-deflection relationships for all tested specimens are presented in Fig. 11, providing comprehensive insights into the structural behavior, stiffness characteristics, ductility, and energy dissipation capacity of different connection configurations under eccentric loading conditions.

Figure 11(a) illustrates the load-deflection behavior of the control specimen S from Group G1. The curve demonstrates typical brittle punching shear failure characteristics with limited ductility. The specimen exhibits a relatively linear elastic response up to the cracking load, followed by a short post-cracking phase before reaching the ultimate load of 13 kN at approximately 2 mm deflection. The sharp drop in load-carrying capacity immediately after peak load indicates sudden punching failure with minimal warning, confirming the absence of any connection enhancement mechanisms. The limited deflection capacity and abrupt failure mode highlight the vulnerability of unreinforced CFST-slab connections under eccentric loading.

Figure 11(b) presents the load-deflection curves for Group G2 specimens with single-row welded bar connections. Both specimens S-1WR8 and S-1WR10 demonstrate substantial improvements in stiffness, strength, and ductility compared to the control specimen S. Specimen S-1WR8 (D8 bars) exhibits enhanced performance with a more gradual post-cracking response, reaching an ultimate load of 21 kN at approximately 4 mm deflection. Specimen S-1WR10 (D10 bars) shows further improvement with a stiffer initial response and higher ultimate capacity of 26 kN achieved at approximately 5 mm deflection. The curves indicate that increasing the welded bar diameter significantly enhances both the load-carrying capacity and deformation capacity. The more gradual post-peak softening behavior compared to specimen S demonstrates improved ductility and energy dissipation, with the welded bars providing effective load transfer and preventing sudden punching failure.

Figure 11(c) displays the load-deflection relationships for Group G3 specimens featuring double-row welded bar configurations. Specimen S-2WR10 demonstrates substantial improvement with an ultimate load of 38 kN at approximately 6 mm deflection, showing enhanced stiffness and ductility compared to single-row configurations. Specimen S-2WR10-N, with slab reinforcement positioned near the welded rows, exhibits further improvement with an ultimate capacity of 45 kN and extended deflection capacity reaching approximately 7 mm. The curve shows improved initial stiffness and more gradual load degradation in the post-peak region. Specimen S-2WR10-A3, combining welded bars with four bolts, achieves the highest performance within the group with an ultimate load of 56 kN at approximately 8 mm deflection. This hybrid configuration demonstrates superior stiffness, strength, and ductility, with the most extensive post-peak plateau indicating excellent energy dissipation capacity. The progressive enhancement from S-2WR10 to S-2WR10-A3 clearly demonstrates the synergistic benefits of combining welded bars with bolts and optimizing reinforcement positioning.

Figure 11(d) illustrates the influence of bolt embedded length through the load-deflection curves of Group G4 specimens. Specimen S-A1 (16 mm embedded length) shows moderate improvement over the control specimen, reaching an ultimate load of 20 kN at approximately 4 mm deflection. Specimen S-A2 (32 mm embedded length) demonstrates enhanced performance with an ultimate capacity of 27 kN achieved at approximately 5 mm deflection, exhibiting improved initial stiffness and more ductile post-peak behavior. Specimen S-A3 (48 mm embedded length) achieves the highest performance with an ultimate load of 33 kN at approximately 6 mm deflection. The curves clearly show that increasing bolt embedded length progressively enhances both the elastic stiffness and ultimate capacity. The post-peak behavior becomes increasingly ductile with longer embedded lengths, indicating that adequate bolt anchorage is essential for achieving effective load transfer and preventing premature connection failure under eccentric loading.

Figure 11(e) presents the load-deflection relationships for Group G5 specimens, examining different reinforcement configurations with constant bolt embedded length. Specimen S-A3-W (wide reinforcement) shows an ultimate load of 26 kN at approximately 5 mm deflection, with a relatively ductile post-peak response but lower capacity compared to the baseline S-A3. Specimen S-A3-M (steel mesh) demonstrates significant improvement with an ultimate capacity of 35 kN achieved at approximately 7 mm deflection, exhibiting enhanced stiffness and a more gradual post-peak degradation. The curve indicates that the steel mesh effectively controls crack propagation and maintains load-carrying capacity over larger deformations. Specimen S-A3-D (upper reinforcement) achieves the highest performance with an ultimate load of 39 kN at approximately 8 mm deflection, demonstrating superior initial stiffness and excellent ductility. The extended post-peak plateau indicates substantial energy dissipation capacity, confirming that strategic placement of upper reinforcement combined with adequate bolt anchorage represents an optimal configuration for eccentric loading conditions.

Figure 11(f) displays the load-deflection curves for Group G6 specimens with C-shaped connection configurations. Specimen S-C16-A2 (24 mm embedded length) achieves an ultimate load of 42 kN at approximately 7 mm deflection, demonstrating high initial stiffness and substantial ductility. The curve shows a well-defined post-peak plateau with gradual strength degradation, indicating effective energy dissipation. Specimen S-C16-A3 (48 mm embedded length) exhibits the highest overall performance among all tested specimens, reaching an ultimate capacity of 49 kN at approximately 8 mm deflection. The curve demonstrates superior elastic stiffness, extended plastic deformation capacity, and excellent post-peak ductility. The C-shaped connections provide effective mechanical anchorage and load transfer, with the longer embedded length mobilizing greater concrete engagement and enhancing overall structural performance. The load-deflection behavior confirms that C-shaped connections represent a highly effective solution for CFST-slab connections under eccentric loading, offering exceptional strength, stiffness, and ductility characteristics.

**Fig. 12:**
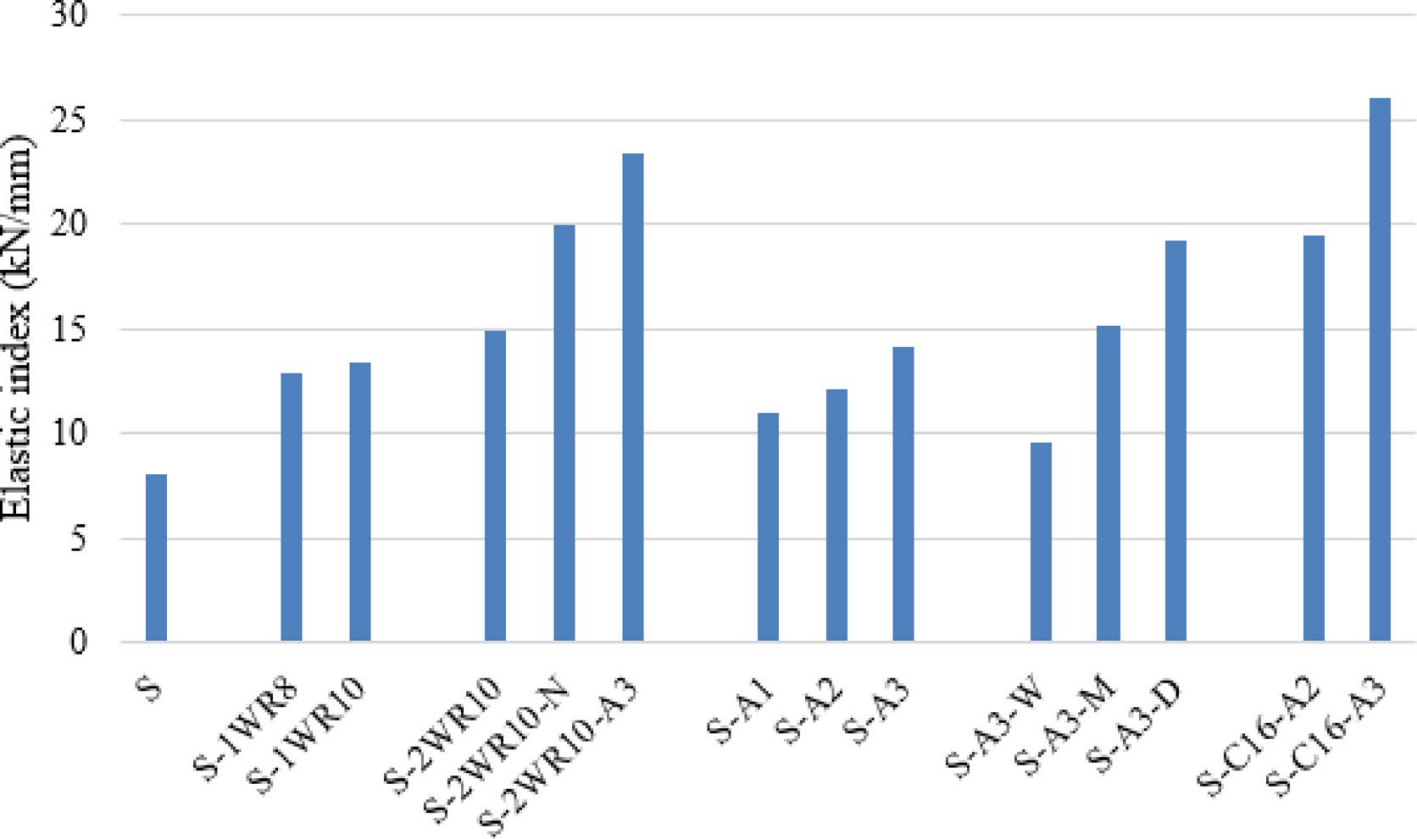
The elastic index for all slabs (kN/mm).

**Fig. 13:**
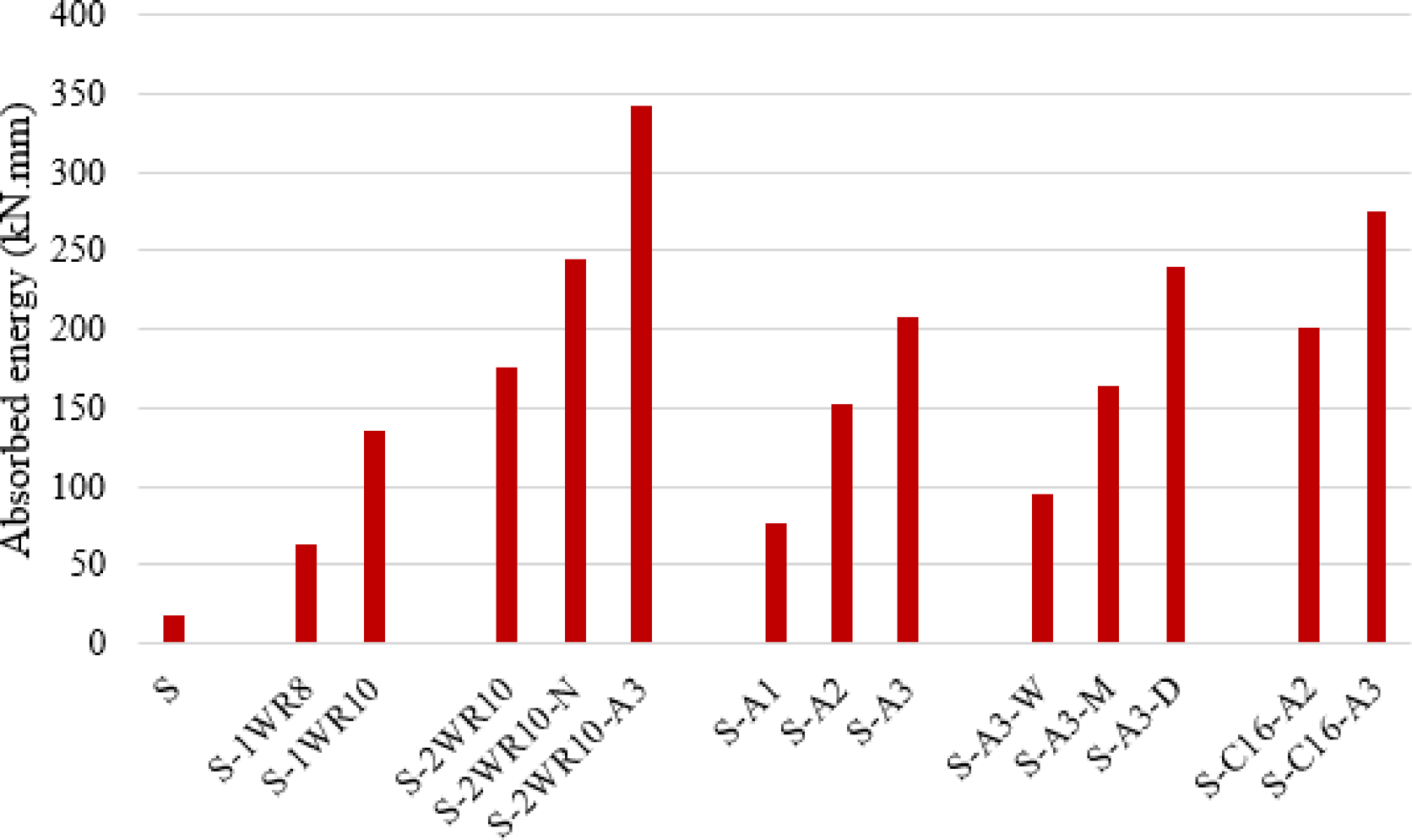
The absorbed energy for all slabs (kN.mm).

Overall, the load-deflection relationships demonstrate that connection configuration significantly influences structural behavior under eccentric loading. The progression from the control specimen to enhanced connection systems shows systematic improvements in stiffness (shown in Fig. 12), strength, ductility, and energy dissipation capacity (shown in Fig. 13), with C-shaped connections and hybrid welded-bolt systems achieving the most favorable performance characteristics.

The absorbed energy was calculated as the area under the load–deflection curve up to the ultimate load, representing the total energy dissipation capacity of the specimen prior to failure. Numerical integration of the experimental load–deflection response was used to determine this value. The ductility index was defined as the ratio of the deflection at ultimate load to the deflection at first cracking (µ = Δ_u_/Δ_cr_). This parameter was used to quantify the deformation capacity of the slab–column connection and to compare the relative ductile behaviour of different connection configurations.

In order to evaluate the initial stiffness behavior of the tested specimens, the elastic index was calculated based on the load–displacement response obtained from the experimental results. The elastic index was defined as the ratio between the load corresponding to the first observed crack in the slab and the vertical displacement measured at the same loading stage. This parameter represents the initial elastic stiffness of the CFST column–RC slab connection system prior to significant cracking and nonlinear behavior. Studying the elastic index is particularly important because it provides an indication of the structural rigidity and the ability of the connection configuration to resist deformation under service-level loading conditions. A higher elastic index reflects a stiffer structural response and better crack resistance, which contributes to improved serviceability performance and delayed damage initiation in the slab–column region. Therefore, evaluating the elastic index helps in understanding the influence of different connection configurations on the early-stage structural performance and the overall punching resistance of CFST column–RC slab systems subjected to eccentric loading.

## Conclusion

The experimental investigations provided a detailed understanding of the structural performance of CFST–RC slab connections under eccentric loading. The main conclusions are summarized as follows:

1. The control specimen exhibited brittle punching shear failure, with the steel tube penetrating the slab due to poor bond interaction, confirming the critical role of proper connection detailing.
2. Incorporation of welded bars and bolts substantially enhanced the connection stiffness, strength, and ductility. The two-row welded bar configuration showed significant improvements in load-carrying capacity, while the hybrid welded–bolted (S-2WR10-A3) connection demonstrated the highest overall performance.
3. Increasing bolt embedment length from 16 mm to 48 mm effectively improved anchorage strength and delayed punching shear failure, producing a more ductile load–deflection response.

## Data Availability

The datasets used and/or analyzed during the current study available from the corresponding author on reasonable request.
